# Baltic Sea methanogens compete with acetogens for electrons from metallic iron

**DOI:** 10.1038/s41396-019-0490-0

**Published:** 2019-08-23

**Authors:** Paola Andrea Palacios, Oona Snoeyenbos-West, Carolin Regina Löscher, Bo Thamdrup, Amelia-Elena Rotaru

**Affiliations:** 1 Department of Biology, University of Southern Denmark, Odense, Denmark; 2 Danish Institute for Advanced Study, University of Southern Denmark, Odense, Denmark; 3 Present Address: Department of Microbiology and Molecular Genetics, Michigan State University, Michigan, East Lansing USA

**Keywords:** Water microbiology, Metagenomics, Water microbiology, Metagenomics

## Abstract

Microbially induced corrosion of metallic iron (Fe^0^)-containing structures is an environmental and economic hazard. Methanogens are abundant in low-sulfide environments and yet their specific role in Fe^0^ corrosion is poorly understood. In this study, *Sporomusa* and *Methanosarcina* dominated enrichments from Baltic Sea methanogenic sediments that were established with Fe^0^ as the sole electron donor and CO_2_ as the electron acceptor. The Baltic-*Sporomusa* was phylogenetically affiliated to the electroactive acetogen *S. silvacetica*. Baltic-*Sporomusa* adjusted rapidly to growth on H_2_. On Fe^0^, spent filtrate enhanced growth of this acetogen suggesting that it was using endogenous enzymes to retrieve electrons and produce acetate. Previous studies have proposed that acetate produced by acetogens can feed commensal acetoclastic methanogens such as *Methanosarcina*. However, Baltic-methanogens could not generate methane from acetate, plus the decrease or absence of acetogens stimulated their growth. The decrease in numbers of *Sporomusa* was concurrent with an upsurge in *Methanosarcina* and increased methane production, suggesting that methanogens compete with acetogens for electrons from Fe^0^. Furthermore, Baltic-methanogens were unable to use H_2_ (1.5 atm) for methanogenesis and were inhibited by spent filtrate additions, indicating that enzymatically produced H_2_ is not a favorable electron donor. We hypothesize that Baltic-methanogens retrieve electrons from Fe^0^ via a yet enigmatic direct electron uptake mechanism.

## Introduction

Microbially induced corrosion (MIC) accounts for 20% of the total corrosion costs for the oil and gas industries [1, 2]. Additionally, chemical leaks from corroded waste containers cause health and environmental problems [3, 4]. Previous studies have primarily focused on MIC in sulfide-rich marine environments, where sulfate-reducing bacteria are predominantly causing corrosion [5]. However, in low-sulfide environments such as the Bothnian Bay, Baltic Sea [6], cooperative interactions between microorganisms (i.e. methanogens and acetogens) may be responsible for corrosion of infrastructure [7–10]. Microbial cooperation was proposed to enhance corrosion rates [11], but it is poorly understood. Of the methanogens, particularly *Methanosarcina* have been suggested to play an essential role in corrosion. *Methanosarcina* are frequently associated with corroded Fe^0^-structures from oil, gas, sewage, water storage, and transportation facilities [7–10], and in aquifers where radionuclide-waste is stored underground in steel containers [12].

Nevertheless, of the highly corrosive methanogens described to date (*Methanococcus maripaludis* strains KA1, Mic1c10, MM1264, and *Methanobacterium* strain IM1 [13–16]), none belongs to the genus *Methanosarcina*. Studies of corrosive *Methanococcus* and *Methanobacterium* have shown that H_2_ generated abiotically by Fe^0^ cannot provide sufficient electrons to account for all the methane being produced by these species during growth in the presence of Fe^0^ [13–17]. Consequently, the electron uptake mechanisms proposed for these different methanogenic strains included (1) a direct uptake route [15, 17] or (2) an extracellular enzyme-mediated electron uptake route [18, 19].

(1) Direct electron uptake from Fe^0^ by *Methanobacterium* strain IM1 was suggested as an alternative to abiotic-H_2_ uptake because this strain generated more methane (CH_4_) from Fe^0^ oxidation than a H_2_-utilizing *M. maripaludis* strain [15] with low H_2_-uptake thresholds [20]. IM1 also produced methane when a cathode poised at a potential unfavorable for abiotic H_2_ evolution was the sole source of electrons [17]. However, the mechanism utilized by IM1 for electron uptake directly from Fe^0^ or electrodes is unknown. Also, it is unknown whether other methanogens have similar capabilities.

*Methanosarcina* species were previously shown to carry out Fe^0^-dependent methanogensis presumably by using abiotic-H_2_ evolved at the Fe^0^-surface [21]. However, some *Methanosarcina* cannot use H_2_ [22–26], and even those *Methanosarcina* species that are capable of hydrogenotrophic methanogenesis have high thresholds for H_2_-uptake [27, 28]. Thus, *Methanosarcina* species would not be competitive at abiotic-H_2_ removal from the Fe^0^ surface. Nonetheless, *Methanosarcina-*species dominate on corroded Fe^0^-structures [7–10]. Therefore, we propose that *Methanosarcina* retrieves electrons directly from Fe^0^, extrapolating from recent findings that *Methanosarcina* can retrieve extracellular electrons directly from poised electrodes [29, 30], electrogenic syntrophic partners [31, 32], or electrically conductive particles [31–34]. Plausible scenarios for direct electron uptake in *Methanosarcina* have only recently been substantiated, using comparative transcriptomics [35].

(2) In addition to direct extracellular electron uptake, a second strategy making use of extracellular enzymes to capture electrons from Fe^0^ was described in methanogens [16, 18, 19]. Enzymes like hydrogenases, formate dehydrogenases, or the heterodisulfide reductase supercomplex produced by methanogens can generate H_2_ or formate from Fe^0^-derived electrons [16, 18, 19]. It is not clear whether an extracellular enzyme-dependent strategy would be competitive in corrosive environments. Moribund cells may release enzymes like hydrogenases into their extracellular milieu [19] that can capture electrons freed during Fe^0^-oxidation to reduce protons from solution to H_2_ [36, 37]. H_2_ could then be used non-specifically by a variety of H_2_-utilizers. Sensitive anaerobic enzymes tend to only be stable for a few days outside of the cell [38]. However, Fe^2+^ released during the corrosion process may further stabilize these enzymes [39]. Hydrogenase-mediated H_2_ production at the Fe^0^ surface appears to generate sufficient H_2_ for the growth of hydrogenotrophic methanogens [16, 19].

Corroded infrastructure often harbors both *Methanosarcina-*methanogens and acetogens, where *Methanosarcina* is thought to play a role in Fe^0^-corrosion [7–10]. However, *Methanosarcina’s* role was assumed to be indirect and dependent on cooperation with other corrosive organisms. For example, *Methanosarcina* was proposed to utilize acetate produced by acetogens actively corroding Fe^0^. In this study, we investigated the theory that acetoclastic methanogens like *Methanosarcina* require cooperative interactions with acetogens to corrode Fe^0^. We used Fe^0^ to enrich for *Methanosarcina* species from sediments collected off the coast of Bothnia. Molecular and physiological tests were used to investigate the role of methanogens and their possible synergy with co-occurring microbes during Fe^0^ corrosion. We present evidence that Baltic-methanogens perform Fe^0^-dependent methanogenesis and compete with acetogens for access to Fe^0^. Specific inhibition experiments indicate that two different mechanisms for Fe^0^-dependent electron uptake by Baltic-acetogens and methanogens are feasible.

## Materials and methods

### Baltic-Sea-enrichment cultures

We collected sediment cores from the Bothnian Bay, Baltic Sea at a water depth of 15 m (65°43.6′N and 22°26.8′E; station RA2) during August 2014 [40]. The sediment had a temperature of 15 °C and an in situ salinity of 0.5. The mineral content was low in insoluble manganese oxides, high in insoluble FeS, and high in crystalline iron oxides, such as semiconductive goethite or conductive magnetite, as previously described [40].

Enrichment cultures were prepared using sediment from the methanogenic zone (30–36 cm) under aseptic and anoxic conditions as previously described [40], but with the addition of 100 g/L iron granules, and exclusion of sulfide as a reducing agent, which was instead replaced with an additional 2 mM cysteine (*c*_f_ _=_ 3 mM). We prepared all subsequent transfers in 50 mL blue chlorobutyl-rubber-stoppered glass vials with an anoxic headspace of CO_2_/N_2_ (20:80, v/v). For all enrichment incubations, we used a DSM120-modified medium (modifications: 0.6 g/L NaCl, without casitone, sodium acetate, methanol, or Na_2_S×9H_2_O). For Fe^0^ incubations, we added to the media as sole electron donor iron granules (99.98%, ThermoFisher, Germany) or iron coupons (3 cm × 1 cm × 1 mm). Other electron donors tested included H_2_ (ca. 1.5 atm) and acetate (10 mM).

All culture experiments were carried out in at least triplicate and sometimes up to 10 replicates. As soon as methane production reached stationary phase, we transferred cells into fresh media with Fe^0^. Cultures were shaken vigorously to remove cells from Fe^0^-granules. We then used 10–20% of the dispersed cells to inoculated fresh Fe^0^-media for subsequent transfers. The temperature used for all incubations was 20 °C. To reach stationary, initial enrichments required circa 6 months, whereas later transfers (T3) took circa 3 months and most recent incubations (T10) took only 1–2 months.

Abiotic control experiments lacked cells. However, we used the same Fe^0^-media and incubation conditions as done for experiments with cells.

Inhibition experiments were carried out by the addition of inhibitors specific for methanogens or bacteria. A methanogenesis inhibitor (2 mM 2-bromoethane sulfonate [41]) was added to the culture media to generate a bacteria-only culture. In order to generate a methanogen-only culture, we added a mixture of antibiotics (200 µg/mL kanamycin and 100 µg/mL ampicillin) to the culture media. Experiments with inhibitors were run parallel to controls lacking inhibitors.

Spent filtrate addition experiments were carried out by the addition of 1 mL spent media from a stationary culture to a new culture as described previously [40]. The spent media of a *Sporomusa* acetogen and a *Methanococcus* methanogen were previously shown to contain electroactive enzymes, which retrieve electrons from Fe^0^ or electrodes for proton reduction to H_2_ [42]

Downstream analyses, DNA extractions, substrate evaluations, and microscopy were performed after the fifth consecutive transfer on Fe^0^.

### Chemical analyses

To determine methane and H_2_ concentrations, we used a Trace 1300 gas chromatography system (Thermo Scientific, Italy) equipped with a thermal conductivity detector (TCD), an injector operated at 150 °C and a detector at 200 °C with 1.0 mL/min reference gas flow. The oven temperature was constant at 70 °C. A TG-BOND Msieve 5A column (Thermo Scientific; 30-m length, 0.53-mm i.d., and 20-μm film thickness) was used with argon as the carrier gas with a set flow at 25 mL/min. The GC was controlled and automated with the Chromeleon software (Dionex, Version 7). Using this setup, the minimum detection limit for methane and H_2_ was 5 µM.

For determination of acetate concentrations, we used a Dionex ICS-1500 Ion Chromatography System (ICS-1500) equipped with the AS50 autosampler, and an IonPac AS22 column coupled to a conductivity detector (31 mA). For separation of volatile fatty acids, we used 4.5 mM Na_2_CO_3_ with 1.4 mM NaHCO_3_ as eluent. The run was isothermic at 30 °C with a flow rate of 1.2 mL/min.

For determination of ferrous iron (Fe^2+^) produced by Fe^0^-oxidation in our cultures, we dissolved Fe^2+^ in 0.67 M HCl (containing 0.67 M hexamethylenetetramine to avoid dissolution of metallic iron) and quantified Fe^2+^ concentrations colorimetrically with the ferrozine assay [43].

For elemental analyses of the gray-black crust that formed on Fe^0^-coupons after 2 months when cells were present, the crust was scraped off the Fe^0^-coupons and dried in an anoxic glove box. Mass spectrometry informed on the content of carbonate and organic carbon. For total reduced inorganic sulfur determination (including iron monosulfides, pyrite, and S^0^) we performed hot chromium distillation [44]. The organic carbon quantification took place after acidification with HCl. We calculated the value for the carbon in carbonates by subtracting the organic-carbon values remaining after acidification from the total unacidified carbon (C-total).

### DNA purification from microbial enrichments

DNA purification was performed using a combination of two commercially available kits; the MasterPure™ Complete DNA and RNA Purification Kit (Epicenter, Madison, Wi, USA), and the Fast Prep spin MP^TM^ kit for soil (Mobio/Qiagen, Hildesheim, Germany). For DNA extraction, we pelleted 10 mL cells, either by harvesting an entire culture grown on Fe^0^ or by removing 10 mL from a larger volume after vigorous shaking the Fe^0^-cultures in order to detach cells from the Fe^0^-surface. We used an Epicenter kit to initiate the DNA extraction with the following modifications to the manufacturer’s protocol: a three-fold higher concentration of proteinase K was added to ensure cell lysis, and a prolonged incubation time at 65 °C was performed until the color of the samples changed from black to brown (the brown pellet gave higher DNA extraction efficiencies). After DNA extraction, we used the Fast Prep spin MP^TM^ kit for soil to carry out RNase treatment and protein precipitation. An advantage of this kit is that it allowed removal of the high iron content, while simultaneously purifying DNA on a binding matrix. Quality and quantity of genomic DNA were determined by electrophoresis on a 1% agarose gel and by UV spectrophotometry on a mySPEC spectrophotometer (VWR^®^, Germany).

### Metagenome analyses

After a single whole-genome amplification cycle, random shotgun metagenome sequencing was performed commercially (Macrogen/Europe) using the Illumina HiSeq2500 platform. We merged the unassembled DNA sequences, checked for quality, and annotated using the Metagenomics Rapid Annotation (MG-RAST) server (vs. 4.03) with default parameters [45]. Shotgun metagenome sequencing resulted in 10,739 high-quality assembled reads of a total of 10,749 with an average length of 167 bp. We obtained metagenome taxonomy information using the databases available in MG-RAST, including Silva [46], RDP [47], Greengenes [48], and RefSeq [49]. For the metagenome taxonomy, the horizontal asymptote of the rarefaction curve indicated complete coverage of the prokaryotic diversity in these samples. For metagenome taxonomy analyses, we used the default MG-RAST cutoff parameters: *e*-value of 1E–5, a minimum identity of 60%, and a maximum alignment length of 15 bp. The metagenome data is available at MG-RAST with this ID: MGM4796413.3.

### 16S rRNA gene sequence analyses

General *Archaeal* and *Bacterial* primers (Table 1SM) were used to perform PCR amplification of the 16S rRNA gene from the isolated DNA. PCR reactions contained in a final volume of 50 μL, 1.5 mM MgCl_2_, 0.2 mM dNTPs, 0.2 μM of each primer, and 1U Promega Taq polymerase, and 10x PCR reaction buffer. PCR reactions included an initial denaturation step at 94 °C for 10 min; then 35 cycles of denaturation at 94 °C for 30 s, annealing at the specific annealing temperature for the primer pair (Table 1SM) for 30 s, and extension at 72 °C for 90 s; and a final extension cycle at 72 °C for 10 min. Next, we cloned PCR products with the TOPO^®^ TA Cloning^®^ Kit for Sequencing (Invitrogen, Carlsbad, CA, USA). PCR products from individual clones were amplified with M13-vector primers and sent to the Institute of Clinical Molecular Biology in Kiel for Sanger sequencing. Sequences were analyzed using the Geneious^®^ software package, version 11.0.4 [50], and compared against the NCBI GenBank DNA database using BLAST. Consensus sequences for *Archaea* and *Bacteria* (97% identity) were assembled using ClustalW implemented within Geneious. Consensus 16S rRNA gene sequences were used to construct maximum likelihood phylogenetic trees in Geneious using RaxML [51]. We deposited sequences in GenBank, under the accession number: MK433201.

### Quantitative PCR

Extracted DNA was used for 16S rRNA gene quantification via qPCR with specific *Sporomusa*, *Methanosarcina*, and general *Bacteria* primers (Table 1SM). For quantification of the members within the corrosive Baltic Sea community, we carried out qPCR assays on duplicate cultures harvested at different times during their growth; 18 days (T10) and 60 days (T9). For each biological replicate, we run quadruplicate qPCR reactions alongside quadruplicate standards (10^1^–10^8^ 16S rRNA gene copies per ml). All standards were prepared as previously described [40, 52].

We prepared the qPCR reaction mix as described before in a final volume of 25 µl of which 10 µl were a 5Prime Hot Master Mix, 0.25 µl BSA (stock 10 mg/ml), 1 µl or forward and reverse primer (10 µM stock each) and 1 µl of a template [40]. The qPCR amplification ran as follows: 2 min hot start at 94 °C, 1 min denaturation at 94 °C, 1 min at the annealing temperature appropriate for the primer pair used (Table 1SM) and 2 min extension at 72 °C. Steps two to four (denaturation, annealing, and extension) were repeated 40 times. The final step was a 10 min elongation step at 72 °C and storage at 4 °C.

### Fluorescence in situ hybridization

To fix cells, we added 2% microscopy grade paraformaldehyde (PFA, 16%) directly to anaerobic cultures and incubated them for 2 h at room temperature. Then all cells were collected with the Fe^0^-granules via centrifugation at 10,000 rpm for 10 min. We gently sonicated (20% intensity; 5 × 5 s) to detach cells from the Fe^0^-granules. Then we collected 50 µL of the resuspended cells by filtration on 0.22 µm filters. We hybridized cells with specific probes (final concentration 5 ng/µL) for *Methanosarcina* (MX821) using the formamide concentration specified in Table 1SM, followed by 2 h hybridization at 46 °C and 15 min. washing at 48 °C [53]. For counterstaining, we incubated the air-dried filters for 3–5 min with a DNA fluorescent stain: 4′,6-diamidino-2-phenylindole (DAPI; 1 µg/ml).

### Epifluorescence microscopy

To confirm the presence or absence of methanogens, we also used their natural autofluorescence due to coenzyme F_420_ and visualized the cells on an epifluorescence microscope equipped with a 420 nm excitation filter as previously described [54]. To visualize cells, we used an upright epifluorescence microscope from Zeiss (Axioscope A1) equipped with a Cy3 (excitation 549 nm, emission 562 nm), a DAPI (excitation 359 nm, emission 461 nm), and an F_420_-filter set (excitation 420 nm, emission 480 nm). For image acquisition, we used a digital CCD camera (Axiocam) controlled by an Axiovision vs. 4.7 software.

### Scanning electron microscopy and sample preparation

We carried out SEM visualization on cells from the fifth transfer that have been growing on Fe^0^-coupons for circa 3 months. We removed the excess culture media and directly fixed cells attached to the Fe^0^-coupon in the anaerobic culture vials using a mix of 2.5% (v/v) glutaraldehyde in 0.1 M phosphate buffer (pH 7.3). Cells were incubated at 4 °C for 12 h, washed in phosphate buffer, dehydrated with anoxic ethanol at increasing concentrations (35%, 50%, 70%, 80%, 90%, 95%, 100%, and three times in 100% v/v; each step for 10 min). The Fe^0^-coupons were then chemically dried with hexamethyldisilazane for 30 min [55] and traces of hexamethyldisilazane were evaporated under N_2_. We stored dried-out Fe^0^-coupons in the culture bottle under N_2_-gas before electron microscopy. Scanning electron microscopy (SEM) was performed with a FESEM Magellan 400 at 5.0 kV at the microscopy facility of the University of Massachusetts, Amherst, USA.

## Results and discussion

Previous studies of corrosive non-sulfidic environments attributed corrosion to syntrophic interactions between acetogens (e.g. *Clostridium*) and acetate-utilizing methanogens (*Methanosarcinales*) [7–10]. Here we challenge this assumption and demonstrate that acetogens and methanogens compete for electrons from Fe^0^, rather than operating cooperatively.

A corrosive community became enriched from methanogenic sediment collected off the Swedish coast using Fe^0^ as the sole electron donor and CO_2_ as the sole electron acceptor. After the establishment of original slurries on Fe^0^ (25% sediment), cultures were transferred sequentially into fresh Fe^0^-containing media using a 10–20% inoculum for the next 3 years.

**Fig. 1:**
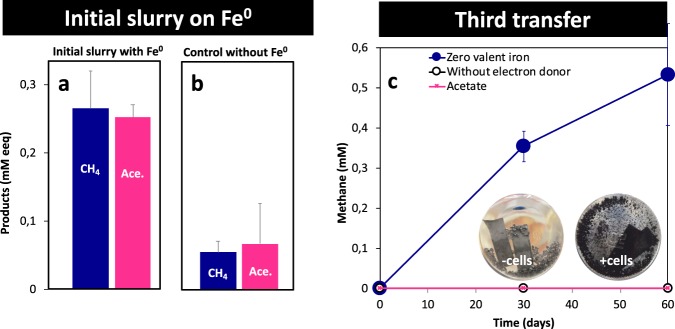
Initial slurries established from methanogenic sediments collected off the coast of the Baltic Sea and third transfer incubations with Fe^0^ or acetate as electron donors vs. parallel control incubations without electron donors. **a** Slurries incubated with Fe^0^ or **b** without Fe^0^ (*n* = 3). Electron conversions into products (methane and acetate) are presented as mM electron equivalents (mM eeq) taking into account that a mol methane/acetate requires 8 mols electrons according to the reactions: CO_2_ + 8e^-^ + 8H^+^ → CH_4_ + 2H_2_O (methanogenesis) and 2 CO_2_ + 8e^-^ + 8H^+^ → CH_3_COOH + 2H_2_O (acetogenesis). **c** Fe^0^-dependent methane production in the third successive passage (sediment-free). However, methane was undetected in electron-donor free controls and when 10 mM acetate replaced Fe^0^ as sole electron donor. The electron donor-free control was used to probe for sediment carryover substrates (**c**-inset). A gray-black corrosion product was observed only in the presence of cells and not in their absence (**c**-inset)

Original slurries from Baltic Sea sediments provided with Fe^0^ generated circa five times more methane (Fig. 1a) and four times more acetate (Fig. 1a) than parallel incubations without Fe^0^ (Fig. 1b). Surprisingly, these Fe^0^-containing slurries accumulated acetate (Fig. 1), which was not consumed by the *Methanosarcina* known to harbor these sediments [40].

After three transfers, incubations became sediment-free as determined by (i) visual inspection, (ii) microscopy, and (iii) the fact that the inoculum did not lead to product formation from carryover of electron donors in incubations without external electron donors (Figs. 1c and 1SM). To verify for acetate utilization by acetoclastic methanogens, Fe^0^ was replaced with 10 mM acetate as the sole substrate for growth. Acetate did not lead to methane production after 2 months of incubation, whereas Fe^0^ did (Fig. 1c), indicating that enriched Baltic-methanogens became adjusted to Fe^0^-dependent methanogenesis, and were not capable of acetoclastic methanogenesis during the given time frame of 2 months.

These sediment-free cultures formed a black crust on the surface of the metal, which was absent in abiotic incubations (Fig. 1c—inset). Under similar conditions (non-sulfidic, carbonate-buffered, pH~7), Fe^0^ is oxidized primarily into Fe^2+^-carbonates such as siderite [15, 56, 57]. Microorganisms like methanogens or acetogens accelerate Fe^0^-oxidation to Fe^2+^ via processes that are energetically more favorable than the abiotic reaction (Table 1). During the eighth transfer, we removed and analyzed the gray-black precipitate formed on Fe^0^ by the Baltic Sea methanogenic community. We determined that the precipitate had a high carbonate content (ca. 50% by weight in FeCO_3_ equivalents), but low reduced inorganic sulfur content (ca. 0.1%; including sulfides), consistent with the formation of iron carbonates, like siderite. The remaining organic carbon content was ~1%.

**Table 1:** Possible reactions occurring at the Fe^0^ surface in non-sulfidic carbonate-buffered media

Process	Reaction	Delta *G*^0′^
Abiotic Fe^0^ dissolution in carbonate- buffered systems	${\mathrm{Fe}}^0 + {\mathrm{HCO}}_3^ - + {\mathrm{H}}^ + \to {\mathrm{FeCO}}_{\mathrm{3}}{\mathrm{ + 2H}}^ + {\mathrm{ + 2e}}^ -$	−79.9 kJ/mol Fe^0^-used
Abiotic H_2_—evolution	$2{\mathrm{H}}^ + + 2{\mathrm{e}}^ - \to {\mathrm{H}}_2$	
Methanogenesis from Fe^0^	$4{\mathrm{Fe}}^0 + {\mathrm{CO}}_2 + 4{\mathrm{HCO}}_3^ - + 4{\mathrm{H}}^ + \to {\mathrm{CH}}_4 + 4{\mathrm{FeCO}}_3 + 2{\mathrm{H}}_{\mathrm{2}}{\mathrm{O}}$	−111.5 kJ/mol Fe^0^-used
Acetogenesis from Fe^0^	$4{\mathrm{Fe}}^0 + 2{\mathrm{CO}}_2 + 4{\mathrm{HCO}}_3^ - + 4{\mathrm{H}}^ + \to {\mathrm{CH}}_{\mathrm{3}}{\mathrm{COOH}} + {\mathrm{4FeCO}}_{\mathrm{3}} + {\mathrm{2H}}_{\mathrm{2}}{\mathrm{O}}$	−97 kJ/mol Fe^0^-used

### Enhanced Fe^0^-oxidation to Fe^2+^ by Baltic Sea acetogens and methanogens

**Fig. 2:**
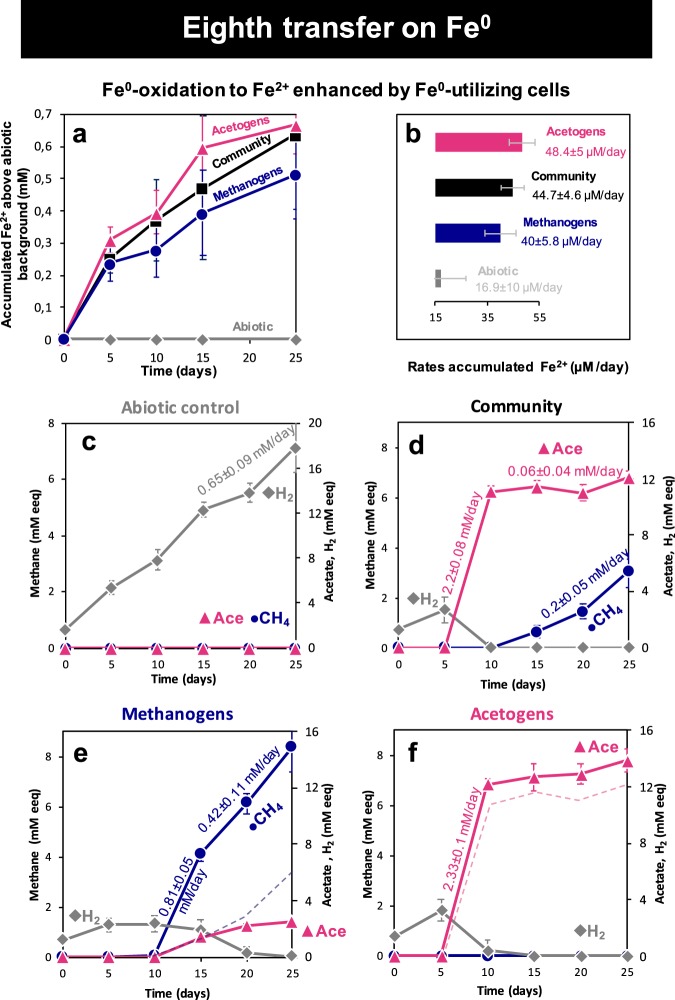
Products of Fe^0^ exposure to microorganisms from the Baltic Sea. **a** Cells enhanced Fe^0^ oxidation to Fe^2+^ above the background observed in cell-free (abiotic) controls. **b** Absolute rates of Fe^2+^-yield over 25 days in the presence or absence of cells. **c** In abiotic controls, electrons were recovered as H_2_. **d** The entire community grown on Fe^0^ for eight transfers recovered electrons as methane and acetate. **e** Inhibition of all bacteria (including acetogens) with a mixture of antibiotics led to enhanced methanogenesis (filled line) compared to the mixed-community (dotted line). **f** Specific inhibition of methanogens with BES, enhanced acetogenesis (filled line) compared to the mixed-community (dotted line) (*n* > 5)

Fe^0^ corrosion was assessed using ferrous iron (Fe^2+^) accumulation as a proxy for corrosion (Fig. 2a, b), as done before, particularly in studies documenting corrosion by methanogens [13–15, 58, 59].

In our incubations, the corrosive microbial community started Fe^0^-oxidation to Fe^2+^ immediately and persisted for circa 25 days (Fig. 2a). The presence of cells led to a tripling of the Fe^2+^ yield per day (44.7 ± 4.6 µM/day) compared to the background Fe^2+^ yield observed in abiotic controls (16.9 ± 10 µM/day) (Fig. 2b). The daily increase of Fe^2+^ in the presence of a Baltic-community indicates that the community was more corrosive than abiotic controls.

Fe^0^-corrosion under non-sulfidic, carbonate-rich conditions in the absence of electron acceptors other than CO_2_ can be attributed to two possible metabolisms: Fe^0^-dependent methanogenesis or Fe^0^-dependent acetogenesis. Theoretically, Fe^0^-dependent methanogenesis is energetically more favorable than Fe^0^-dependent acetogenesis, but both are energetically more favorable than abiotic Fe^0^-dissolution (Table 1). Therefore, to better understand the interplay between acetogens and methanogens within the corrosive microbial community we (1) monitored product evolution and (2) inhibited various metabolic groups in order to determine the corrosive potential of each surviving group.

### Electron recoveries from Fe^0^

After eight transfers on Fe^0^-containing medium, the Baltic Sea corrosive enrichment cultures exhibited a quick Fe^0^-dependent acetogenic phase (days 5–10) followed by a slow methanogenic phase (days 10–25) (Fig. 2d). During the acetogenic phase, acetogens were able to convert 2.2 ± 0.1 mM/day electron equivalents from Fe^0^ to form acetate (Fig. 2d). As soon as the acetogenic phase stopped, the methanogenic-phase began, and methanogens recuperated 0.2 ± 0.05 mM electron equivalents from Fe^0^ into methane daily. After 25 days, the community routed more electrons into acetate than into methane (Fig. 2d). Thus, it appears that acetogens outcompeted methanogens for access to electrons from Fe^0^.

### Competition between acetogens and methanogens

We evaluated whether methanogens were in competition with acetogens for electrons from Fe^0^ by testing whether methanogens functioned better without acetogens. In order to test this, we inhibited acetogens and other bacteria using antibiotic additions (kanamycin and ampicillin). With acetogens inhibited, methanogens oxidized Fe^0^ to Fe^2+^ at rates above abiotic controls (Fig. 2e) and similar to those observed for the entire community (Fig. 2d). After 15 days, methanogens alone produced six-times more methane (3.8 ± 0.7 mM electron equivalents/eeq CH_4_) (Fig. 2e) than they did when they were co-existing with bacteria in a mixed community (0.6 ± 0.2 mM eeq CH_4_) (Fig. 2d). During the first 5 days of the methanogenic-phase (days 10–15), electron recoveries into methane were higher (0.81 ± 0.06 mM eeq CH_4_) than expected from the rates achievable if cells were dependent on the production of abiotic H_2_ production (0.65 ± 0.09 mM eeq H_2_). Nevertheless, electron recoveries decreased by half (0.42 ± 0.1 mM eeq CH_4_) (Fig. 2e), possibly due to competition for Fe^0^ with acetogens that developed antibiotic resistance and generated 2.5 mM eeq acetate. However, even brief inhibition periods of the acetogens led to significantly higher methanogenic activity (six-fold), indicating that acetogens inhibited methanogenesis on Fe^0^. Sub-optimal methane production suggests that methanogens may experience decreased access to electrons from Fe^0^ due to the competitive exclusion by acetogens.

To test whether methanogens impacted the growth of acetogens, we inhibited methanogens with BES, a methyl–CoA analog [41]. In the presence of the methanogenic inhibitor, methanogens were rendered inactive throughout the incubation (Fig. 2f). Nevertheless, acetogens alone were able to oxidize Fe^0^ to Fe^2+^ (Fig. 2a, b), while producing more acetate (14%; *p* = 0.0001) than they did within the mixed community (Fig. 2f). These data suggest that methanogens constrain the growth of acetogens.

**Fig. 3:**
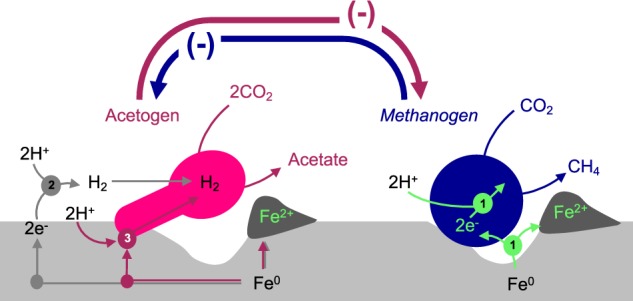
Modeled competitive interaction between Fe^0^-corroding acetogens and Fe^0^-corroding methanogens. (1)— indicates a possible direct mechanism of electron uptake, (2)—indicates a mechanism of electron uptake based on abiotic-H_2_ and (3)—indicates extracellular enzyme-mediated H_2_-evolution

These results indicate that acetogens and methanogens negatively affect one another when competing for Fe^0^ as the sole electron donor (Fig. 3).

### Contrasting mechanisms of electron uptake from Fe^0^ in Baltic-acetogens and methanogens

To investigate the possible mechanisms of electron uptake by Baltic-acetogens and methanogens, we compared electron recoveries in abiotic controls vs. those in the presence of cells. In the absence of cells, Fe^0^ released 0.65 ± 0.09 mM/day electron equivalents as H_2_ (Fig. 2c) continuously for 25 days. When acetogens were present, electron equivalents were recovered 3.5 times faster than expected from abiotic H_2_ (Fig. 2f), excluding electron recovery into biomass unaccounted for during the experiments. Thus, acetogens likely used an alternative mechanism to access electrons from Fe^0^ easily and accelerate acetogenesis.

Acetogens have been shown to use two different mechanisms for electron uptake from Fe^0^ facilitated by enzymes evolving H_2_ [16, 19] or by direct-electron uptake [60–62]. The latter is plausible because several acetogens can grow on electrodes poised at potentials that do not generate abiotic H_2_ [60–62]. On the other hand, previous studies illustrated efficient enzymatic-mediated electron uptake from Fe^0^ using a purified *Clostridium* [FeFe]-hydrogenase, which retrieves electrons directly from Fe^0^ for proton oxidation to H_2_ [36, 37]. Unlike [NiFe]-hydrogenases from methanogens, the [FeFe]-hydrogenases of *Clostridium* are effective at oxidizing H^+^ [63] and quickly evolving H_2_ that could serve as an electron donor for Baltic-acetogens. Therefore, we had to determine whether Baltic-acetogens (i) utilize H_2_, (ii) are stimulated by endogenous enzymes, or (iii) use an alternative direct electron uptake mechanism.

**Fig. 4:**
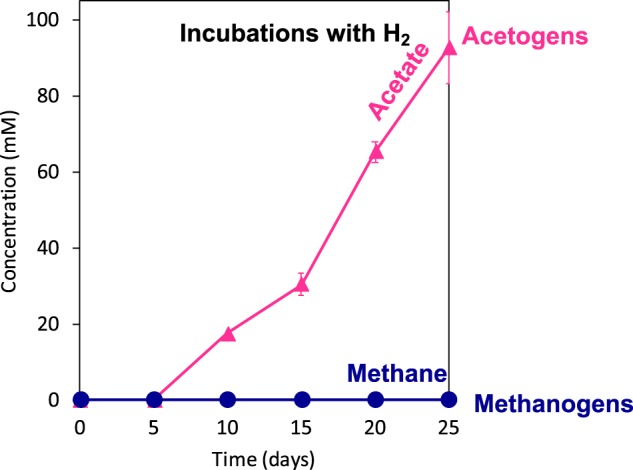
Incubations (9th transfer) replacing Fe^0^ with H_2_ as the electron donor for **a** Baltic-methanogens vs. **b** Baltic-acetogens. To generate culture conditions favorable only for Baltic-methanogens, we added kanamycin and ampicillin to inhibit the acetogens. To generate culture conditions favorable only for Baltic-acetogens, we added BES a specific inhibitor for the methanogens

**Fig. 5:**
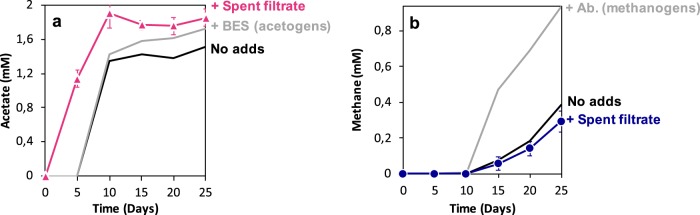
We determined the possible impact of extracellular enzymes/shuttles from spent-filtrate by incubating 10 new cultures in Fe^0^/CO_2_-media spiked with 1 mL spent filtrate (*n* = 10, T8). These incubations were run in parallel with cultures without spent-filtrate (see Fig. 2). For clarity, we separated the acetogens and methanogens into two panels. In the spent-media spiked community we monitored **a** the early on-set for acetogenesis after the addition of spent filtrate to Fe^0^-cultures and **b** inhibition of methanogenesis after the addition of spent-filtrate. Gray lines depict trends for product formation by the acetogens-alone and methanogens-alone after specific inhibition of their competitors. Black lines represent product formation by acetogens and methanogens within the mixed-community

To verify whether Baltic-acetogens could rapidly switch to H_2_ after being adapted to Fe^0^ as the sole electron donor for eight transfers, we incubated the acetogens on H_2_ (after BES-inhibition of methanogens). H_2_-dependent acetogenesis took 5 days to commence, similar to Fe^0^-incubations (Fig. 4). Unlike a 5-day long Fe^0^-dependent acetogenesis (Fig. 2f), H_2_-dependent acetogenesis continued steadily for 20 days (Fig. 4). Although Baltic-acetogens were effective H_2_-utilizers, the rates of abiotic H_2_ formation from Fe^0^ could not explain the tripling in electron recovery rates by acetogens on Fe^0^ (Fig. 2c, f). Therefore, we assumed that extracellular hydrogenases might stimulate electron uptake from Fe^0^ by inducing enzymatic H_2_-formation and subsequently enhancing the rates of acetogenesis from Fe^0^. To determine if such enzymes had a stimulatory effect, we filtered the spent medium of a pre-grown Fe^0^-culture into a fresh culture provided with Fe^0^. If the active enzymes present in the spent-filtrate stimulated the growth of acetogens then the rate of electron recovery from Fe^0^ into acetate would increase. Indeed, spent filtrate stimulated acetogenesis, which started 5 days earlier (Fig. 5a) than it did in Fe^0^-grown or H_2_-grown cultures of Baltic-acetogens. Moreover, acetate recoveries were the highest after the addition of spent filtrate (Fig. 5a) compared to the unamended community (22% increase, *n* = 10, *p* < 0.00001), or unamended acetogens (7% increase; *n* = 10, *p* < 0.02). These results suggest that Baltic-acetogens use an enzyme-mediated mechanism to enhance electron uptake from Fe^0^, similar to other acetogens [19, 55].

Like acetogens, methanogens are believed to retrieve electrons from Fe^0^ via an enzyme-mediated electron uptake mechanism [16, 18, 19, 64] or a poorly understood direct electron uptake system [15, 17]. Thus, to distinguish between these two mechanisms, we tested the impact of H_2_ and spent-media filtrate on the growth of Baltic-methanogens.

Unlike Baltic-acetogens, Baltic-methanogens could not utilize H_2_ for methanogenesis (Fig. 4), also their methane productivity declined after the addition of spent media filtrate (−23%; *n* = 10; *p* < 0.03; Fig. 5b). These results suggest that Baltic-methanogens did not use an enzyme-mediated electron uptake mechanism. These data corroborate with previously published results initiated from the same sediment and in which we observed that *Methanosarcina* was capable of mineral-mediated syntrophy independent of enzymes from spent filtrate additions [40]. Additionally, in the present study we show that Baltic-*Methanosarcina* preferred Fe^0^ as electron donor and were unable to consume acetate or abiotic-H_2_, demonstrating that they were likely retrieving electrons directly from Fe^0^ (Figs. 1b and  4).

### *Sporomusa* and *Methanosarcina* dominate the corrosive microbial community

**Fig. 6:**
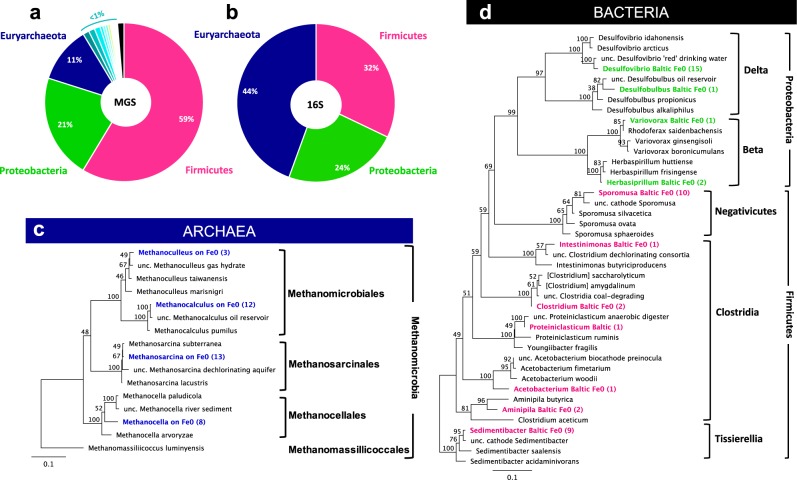
MGS and 16S rRNA gene library studies identified the same representative phylotypes in Baltic Sea corrosive enrichments during sequential transfers. **a** Phylogenetic assignment of *Bacteria* and *Archaea* according to metagenome sequencing (MGS) of duplicate-cultures harvested at the end of transfer #5. **b** Phylogenetic assignment of *Bacteria* and *Archaea* according to 16S rRNA gene libraries assembled from duplicate-cultures harvested at the end of transfer #8; Phylogenetic trees were built using sequences from the 16S rRNA gene libraries. **c** Maximum-likelihood tree showing complete 16S rRNA gene sequences of Baltic-*Archaea*. **d** Maximum-likelihood tree showing 16S rRNA gene sequences of Baltic-*Bacteria*. The numbers in parenthesis represent the number of clones with >97% similarity to each other. The scale bar reflects the mean number of nucleotide substitutions per site as inferred by the RAxML algorithm. The numbers close to the nodes reflect bootstrap values (>70% good support) as inferred from 100 tree iterations using RAxML

16S rRNA gene and metagenome sequence (MGS) analyses of the corrosive community identified *Proteobacteria, Firmicutes*, and *Euryarchaeota* (Fig. 6a, b) as representative phylotypes.

*Proteobacteria* clustered primarily with the genus *Desulfovibrio*. All *Desulfovibrio* sequences were most similar to *D. idahonensis* (97.5% identity; Fig. 6d) which was isolated from a metal(oid) contaminated sediment [65]. Baltic-*Desulfovibrio* were also related (98.5% identity) to a *Desulfovibrio* from a drinking water system contaminated by iron oxides [66]*. Desulfovibrio* species are capable of corrosion [5] under high-sulfate conditions common in marine environments (ca. 28 mM sulfate [67]). However, the methanogenic zone of the Baltic Sea contains no sulfate [6] and thus is a low-sulfide environment (0–2 mM [68]). Therefore, under the low-sulfate conditions in our media (ca. 0.4 mM) *Desulfovibrio* could only (i) use trace sulfate for its metabolism or (ii) ferment dead-biomass organics (e.g. pyruvate, fumarate) alone [69] or syntrophically [70].

**Fig. 7:**
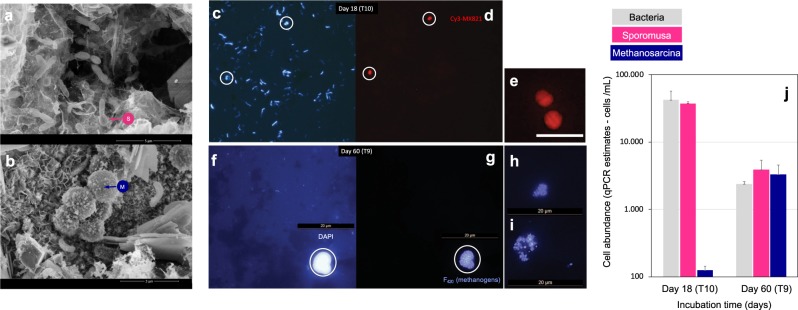
Morphotypes and abundant phylotypes from a Baltic corrosive community. **a** Scanning electron microscopy (SEM) image of a spore-forming curved rod resembling a *Sporomusa* sporulating cell. **b** SEM micrograph of tetrads of cocci resembling *Methanosarcina* and their usual cocci aggregates. SEM was performed at the end of transfer #5 (ca. 3 months). **c** Epifluorescence micrographs of DAPI-stained cells detached from Fe^0^-granules by shaking and sonication during day 18 of transfer #10. We observed two morphotypes: a banana-shaped rod and diplococci. **d** The diplococci were *Methanosarcina* as identified by a specific probe for in situ hybridization (Cy3/red-MX821). **e** Baltic-*Methanosarcina* sometimes also formed tetrads and could never be visualized as single cells in these Fe^0^-dependent cultures. **f** Epifluorescence micrograph of DAPI-stained cells detached from Fe^0^-granules by vigorous shaking during day 60 of transfer #10. Only two morphotypes were observed—banana-shaped rods and cocci joined in large aggregates. **g** *Methanosarcina* formed cocci aggregates which could be detected by their natural F_420_-autofluorescence. No other morphotypes of methanogens could be detected. **h** and **i** *Methanosarcina*- aggregates with various morphologies and compactness. **j** Group-specific qPCR to determine the abundance of *Methanosarcina* and *Sporomusa* after 18 and 60 days of incubation on Fe^0^

Acetogens identified by MGS and 16S rRNA gene libraries belonged to the *Firmicutes* genera *Sporomusa, Clostridium*, and *Acetobacterium* (Fig. 6a, b, d). Spore-bearing curved rods resembling *Sporomusa* were visually observed in our cultures (Fig. 7a). *Sporomusa* dominated (>88%) the bacterial community according to 16S rRNA-gene qPCR analyses, independent of the incubation period (Fig. 7j). The closest isolated relative for our Baltic-*Sporomusa* was *S. silvacetica* (97.4% identity), previously shown to be capable of electroacetogenesis on a cathode at −400 mV vs. SHE [61]. Its closest uncultured relative (99.3% identity) was a *Sporomusa* kenriched on a cathode (Genbank KJ600503 Fig. 6d).

Methanogens identified by MGS and 16S rRNA gene libraries belonged to *Methanomicrobia* represented by the genera *Methanosarcina, Methanocalculus*, and *Methanocella*. Because the Baltic-methanogens could not utilize H_2_ (1.5 atm; Fig. 4) for methanogenesis, we expected *Methanosarcina* to be the dominant member of the methanogenic community. As such, *Methanosarcina* became 26 times more abundant at the end of the incubation period (day 60) than they were during the first stage of incubation (day 18; Fig. 7j). During the early stages of Fe^0^-dependent growth (18 days), *Methanosarcina* cells formed diplococci (Fig. 7d) or tetrads (Fig. 7e), but after 2 months of incubation, multicellular aggregates could be visualized by F_420_-autofluorescence specific for methanogens (Fig. 7g–i). No other methanogenic morphotypes were observed with F_420_-autofluorescence [54], indicating that *Methanosarcina* was the dominant methanogen.

*Methanosarcina* is the only known genus that includes species incapable of methanogenesis from H_2_ [22, 25, 26], acetate [71], or both [23, 24]. Additionally, *Methanosarcina* includes species capable of direct electron uptake from electrodes [29, 30] and other cells either directly [29, 31, 32], or via conductive minerals [29, 31–34, 72]. The closest relative of Baltic-*Methanosarcina* was the non-acetoclastic and non-hydrogenotrophic *M. subterranea* (Fig. 6c). The inability of their closest relative to use H_2_ and acetate aligns with physiological evidence that Baltic-*Methanosarcina* was also incapable of methanogenesis using these substrates (Figs. 1c, 4). Conclusively, Baltic-*Methanosarcina* was unlikely to consume acetate produced by Baltic-acetogens. Hence, our results contest previous suppositions that *Methanosarcina* and acetogens mainly interact syntrophically, via acetate-transfer, within a corrosive microbial community [73]. Instead, we provide evidence that Baltic-*Methanosarcina* were more metabolically active on Fe^0^ in the absence of bacterial partners (Fig. 2e). Indeed, the methanogens appeared to compete with acetogens to access Fe^0^, since Fe^0^-dependent methanogenesis decreased approximately threefold when acetogens were active (Fig. 2d, e).

Moreover, our results indicate that Baltic-*Methanosarcina* might be using a direct mechanism of electron uptake from Fe^0^, since they could not use H_2_, independent of its origin (Figs. 4 and 5b). Extracellular enzyme-facilitated Fe^0^ corrosion has only been demonstrated in *Methanococcus* species [16, 19], while *Methanosarcina* species were thought to retrieve electrons from Fe^0^ via abiotic H_2_ uptake [21]. However, some *Methanosarcina* cannot use H_2_ at all [22–26], while others have high H_2_-uptake thresholds (296–376 nM) [27, 28]. Therefore, when using abiotic or enzymatic H_2_ *Methanosarcina* should be outcompeted by strict hydrogenotrophic methanogens with low H_2_-uptake thresholds (e.g. 6 nM for *Methanobacterium formicicum*) [27, 28], yet this was not the case in our enrichments indicating they may use an alternative electron uptake mechanism. Similar to other studies *Methanosarcina*, rather than strict hydrogenotrophic methanogens dominated the Fe^0^ corroding community, suggesting that *Methanosarcina* can in fact outcompete strict hydrogenotrophic methanogens from a corrosive community. The mechanism of direct electron uptake from Fe^0^ or any other insoluble electron donors employed by *Methanosarcina* is unknown. Extracellular electron uptake in *Methanosarcina* has been examined recently using a comparative transcriptomics approach contrasting *Methanosarcina* provided either with electrons from a current-producing syntrophic partner (electrogenic *Geobacter* [31, 32]) or with H_2_ from a H_2_-producing syntrophic partner (*Pelobacter*) [35]. During extracellular electron uptake from an electrogenic bacterium, *Methanosarcina* up-regulated cell-surface proteins with redox properties, such as cupredoxins, cytochromes, and other Fe-S-proteins [35]. However, the exact role of these redox-active proteins in *Methanosarcina*’s extracellular electron uptake from insoluble extracellular electron donors (Fe^0^, other cells or electrically conductive particles) remains enigmatic and requires future exploration.

## Conclusion

*Methanosarcina* and acetogens often cohabit on the surface of corroded Fe^0^-structures from low-sulfate environments. However, the role of *Methanosarcina* was assumed to be commensal, feeding on the acetate produced by acetogens. Our results demonstrate that Baltic-*Methanosarcina* does not establish a syntrophic partnership with acetogens based on acetate transfer as often reported. Instead, Baltic-*Methanosarcina* and Baltic-*Sporomusa* competed with one another to reclaim electrons from Fe^0^, and each group became favored when specific inhibitors for their competitors were added to the medium. While Baltic-acetogens seem to be stimulated by enzymes/shuttles from spent filtrate, *Methanosarcina* were not. Moreover, Baltic-*Methanosarcina* were unable to utilize acetate and H_2_ as electron donors, suggesting that they may be retrieving electrons directly via a largely unexplored mechanism.

## Supplementary information

Supplementary File
