# Supplementary material for: Baltic Sea methanogens compete with acetogens for electrons from metallic iron
Source: ISME J. 2019 Aug 23;13(12):3011–23. doi: 10.1038/s41396-019-0490-0 (PMC6864099; doi:10.1038/s41396-019-0490-0)
Supplement: Supplementary file 1 — Supplementary File [file 41396_2019_490_MOESM1_ESM.docx]

**Supplementary file: Baltic methanogens compete with acetogens for electrons from metallic iron**

**Authors**: Paola Andrea Palacios^1^, Oona Snoeyenbos-West^1, a^, Carolin Regina Löscher^1,2^, Bo Thamdrup^1^, Amelia-Elena Rotaru^1*^

**Table 1SM.** Primers and probes used in this study

| Primer pair/Probes | Sequence (5’-3’) | Group Coverage* | T_a_/FA % | Ref. |
| --- | --- | --- | --- | --- |
| Univ 27F  UNIV 1492R | AGAGTTTGATCMTGGCTCAG  TACCTTGTTACGACTT | *Bacteria* (78.7%) | 50°C | [1] |
| Arch 4Fa  UNIV 1391R | TCCGGTTGATCCTGCCRG  GACGGGCGGTGTGTRCA | *Archaea* (73.8%) | 50°C | [2] |
| MSc 320F  MSC 828R | GAAACCGYGATAAGGGGA  TAGCGARCATCGTTTACG | *Methanosarcinales* (80%) | 50°C | [3] |
| Univ 27F  Sporo-2 | AGAGTTTGATCMTGGCTCAG  ACCCATCTCTAATCGGTAGC | *Sporomusa* (28.6%) | 56°C (this study) | [4] |
| MX821 – Cy3 | CGCCATGCCTGACACCTAGCGAGC | *Methanosarcina*-genus (87%) | 30% FA | [5] |
| *group coverage verified using SILVA TestPrime vs. 1.0 for primers and SILVA TestProbe vs. 3.0. | | | | |

1. Lane DJ. 16S/23S rRNA sequencing. *Nucleic acid Tech Bact Syst* 1991; 115–175.

2. Robertson CE, Spear JR, Harris JK, Pace NR. Diversity and stratification of archaea in a hypersaline microbial Mat. *Appl Environ Microbiol* 2009; **75**: 1801–1810.

3. Yu Y, Lee C, Kim J, Hwang S. Group-specific primer and probe sets to detect methanogenic communities using quantitative real-time polymerase chain reaction. *Biotechnol Bioeng* 2005; **89**: 670–679.

4. Demaneche S, Sanguin H, Pote J, Navarro E, Bernillon D, Mavingui P, et al. Antibiotic-resistant soil bacteria in transgenic plant fields. *Proc Natl Acad Sci* 2008; **105**: 3957–3962.

5. Raskin L, Stromley JM, Rittmann BE, Stahl DA. Group-specific 16S rRNA hybridization probes to describe natural communities of methanogens. *Appl Environ Microbiol* 1994; **60**: 1232–40.


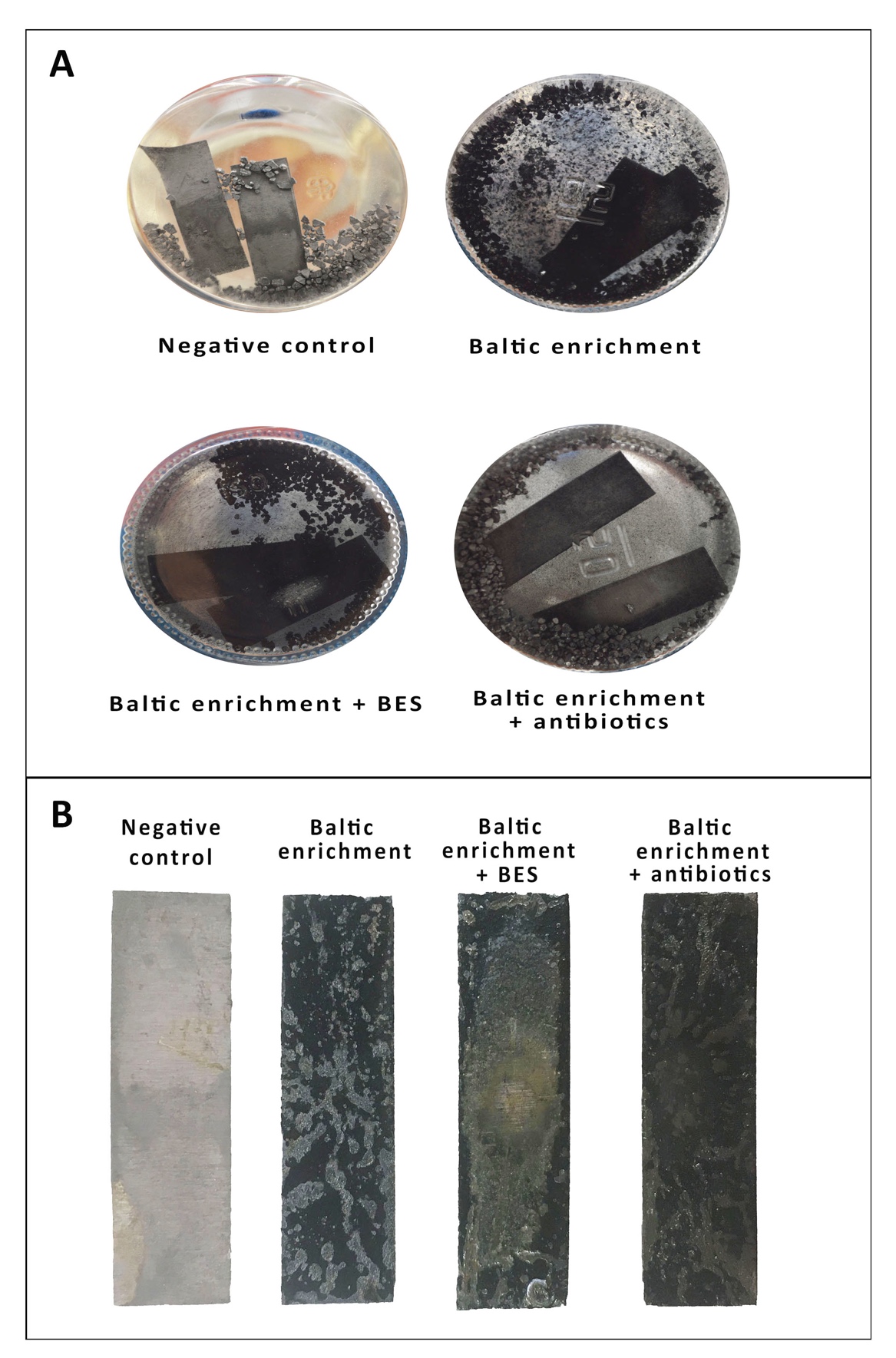
Figure 1SM. Pictures of Fe^0^ coupons with and without cells.
